# Supplementary material for: Bioactivity-Guided Fractionation and NMR-Based Identification of the Immunomodulatory Isoflavone from the Roots of Uraria crinita (L.) Desv. ex DC
Source: Foods. 2019 Nov 3;8(11):543. doi: 10.3390/foods8110543 (PMC6915426; doi:10.3390/foods8110543)
Supplement: Supplementary file 1 [file foods-08-00543-s001.pdf]

Supplementary material

Bioactivity-guided fractionation and NMR-based identification of the immunomodulatory isoflavone from the roots of *Uraria crinita* (L.) Desv. ex DC.

Ping-Chen Tu[a], Chih-Ju Chan[b], Yi-Chen Liu[c], Yueh-Hsiung Kuo[b,d,e], Ming-Kuem Lin[b,*], Meng-Shiou Lee[b,*]

[a] The Ph.D. Program for Cancer Biology and Drug Discovery, China Medical University and Academia Sinica, Taichung 404, Taiwan

[b] Department of Chinese Pharmaceutical Sciences and Chinese Medicine Resources, China Medical University, Taichung 404, Taiwan

[c] Institute of Biomedical Science and Rong Hsing Research Center for Translational Medicine, National Chung-Hsing University, Taichung 402, Taiwan

[d] Department of Biotechnology, Asia University, Taichung 413, Taiwan

[e] Chinese Medicine Research Center, China Medical University, Taichung 404, Taiwan

[*]Corresponding authors.

E-mail addresses: linmk@mail.cmu.edu.tw (M.-K.L.), leemengshiou@mail.cmu.edu.tw (M.-S.L.).

	Table of Contents	Pages
**Figure S1.**	Selected ^{13}C NMR spectrum (acetone-d_6, 125 MHz) of subfraction D-4.	3
**Figure S2.**	Selected HSQC spectrum (acetone-d_6) of subfraction D-4.	4
**Figure S3.**	Selected HMBC spectrum (acetone-d_6) of subfraction D-4.	5
**Figure S4.**	Chromatogram of genistein-containing subfractions D-4 and D-5.	6
**Figure S5.**	The effects of the compounds LA (lupinalbin A), MS (*p*-hydroxybenzoic acid), HA (*p*-hydroxybenzoic acid), DDA (*p*-hydroxybenzoic acid), and ST (a mixture of *β*-sitosterol and stigmasterol) on the production of pro-inflammatory cytokines in LPS-stimulated DCs	7
**Figure S6.**	The effects of the compounds SA (salicylic acid) and VA (vanillic acid) on the production of pro-inflammatory cytokines in LPS-stimulated DCs.	8

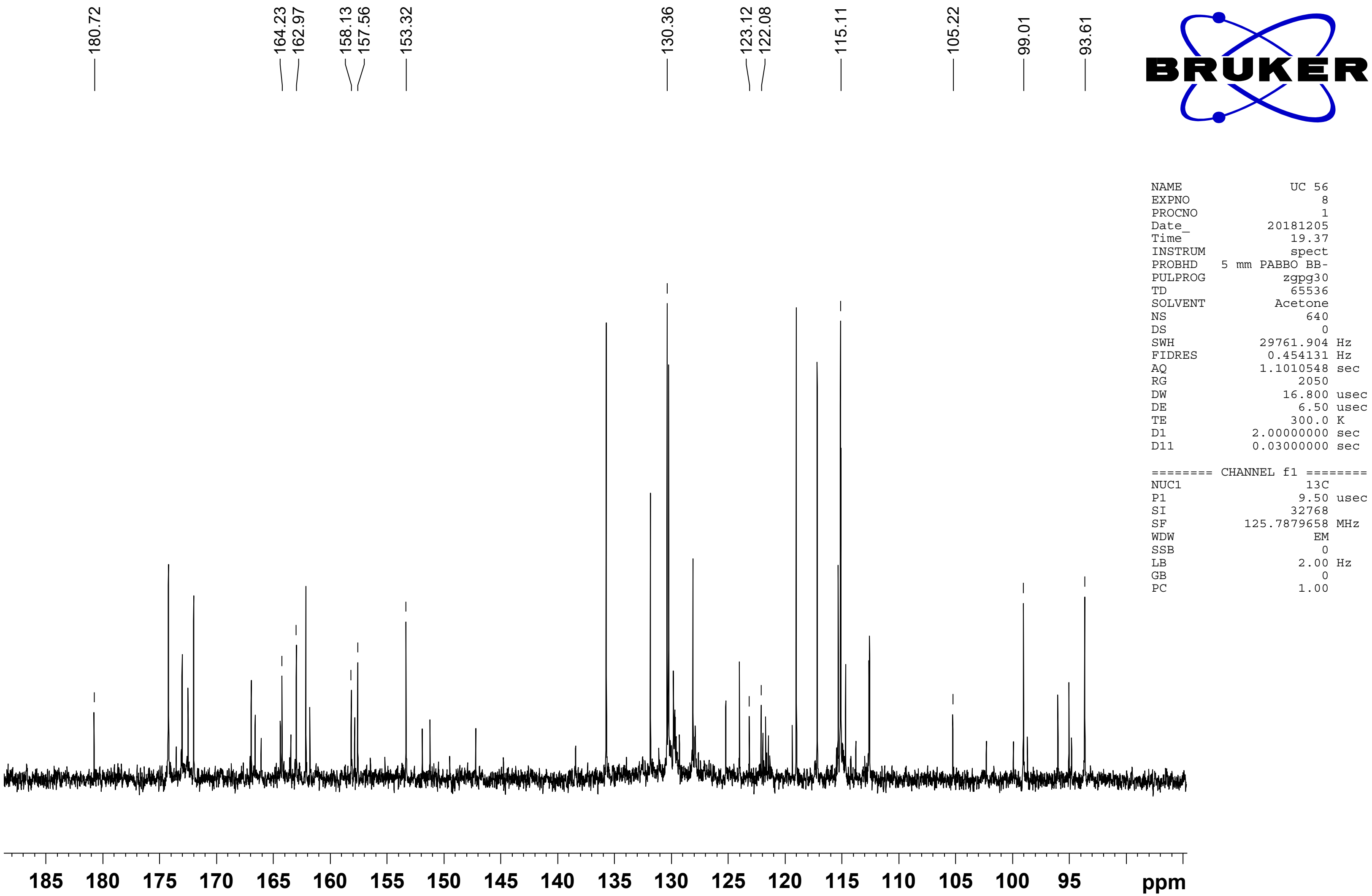

Fig. S1 Selected ^{13}C NMR spectrum (acetone-d_6, 125 MHz) of subfraction D-4.

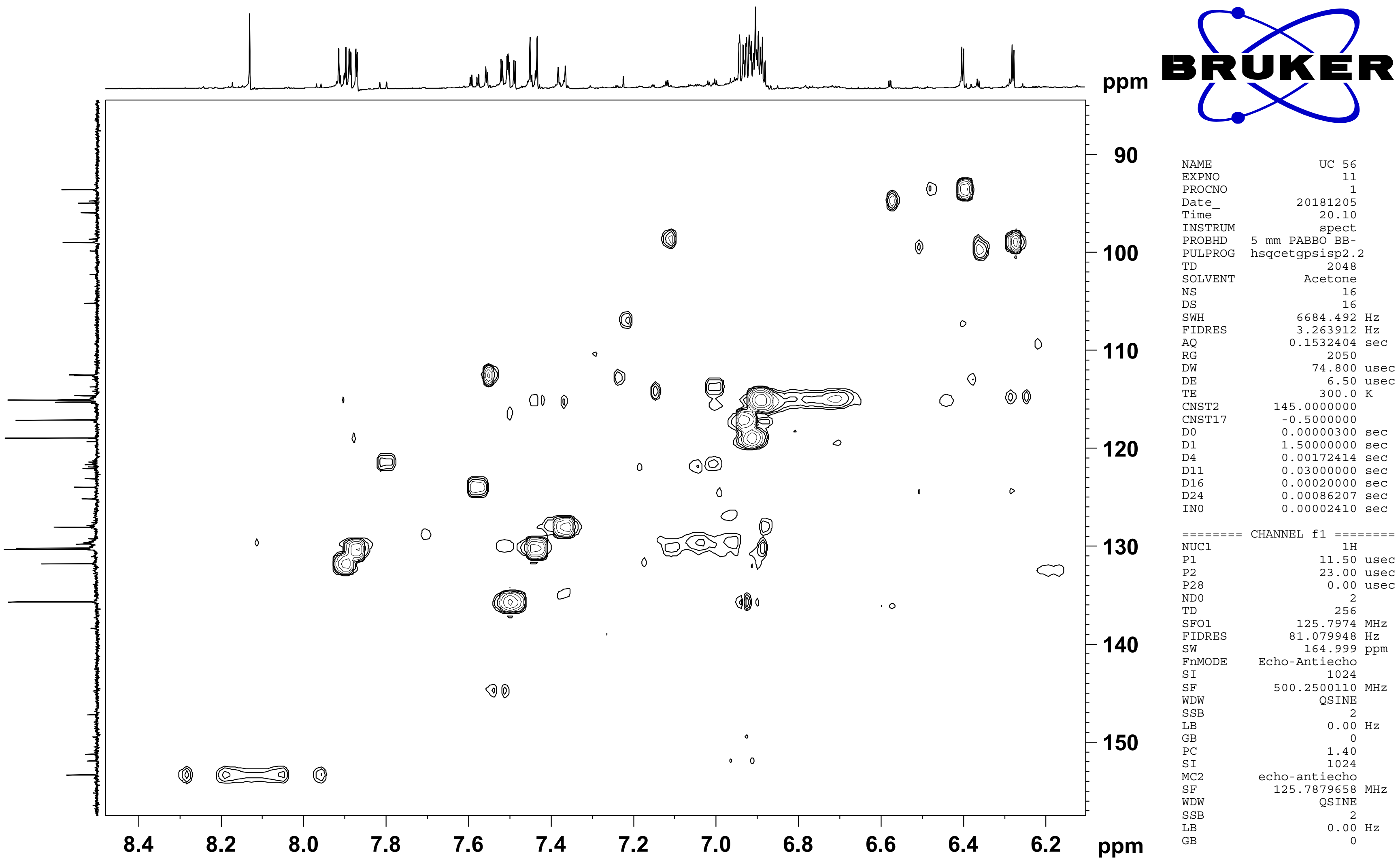

Fig. S2 Selected HSQC spectrum (acetone-d_6) of subfraction D-4.

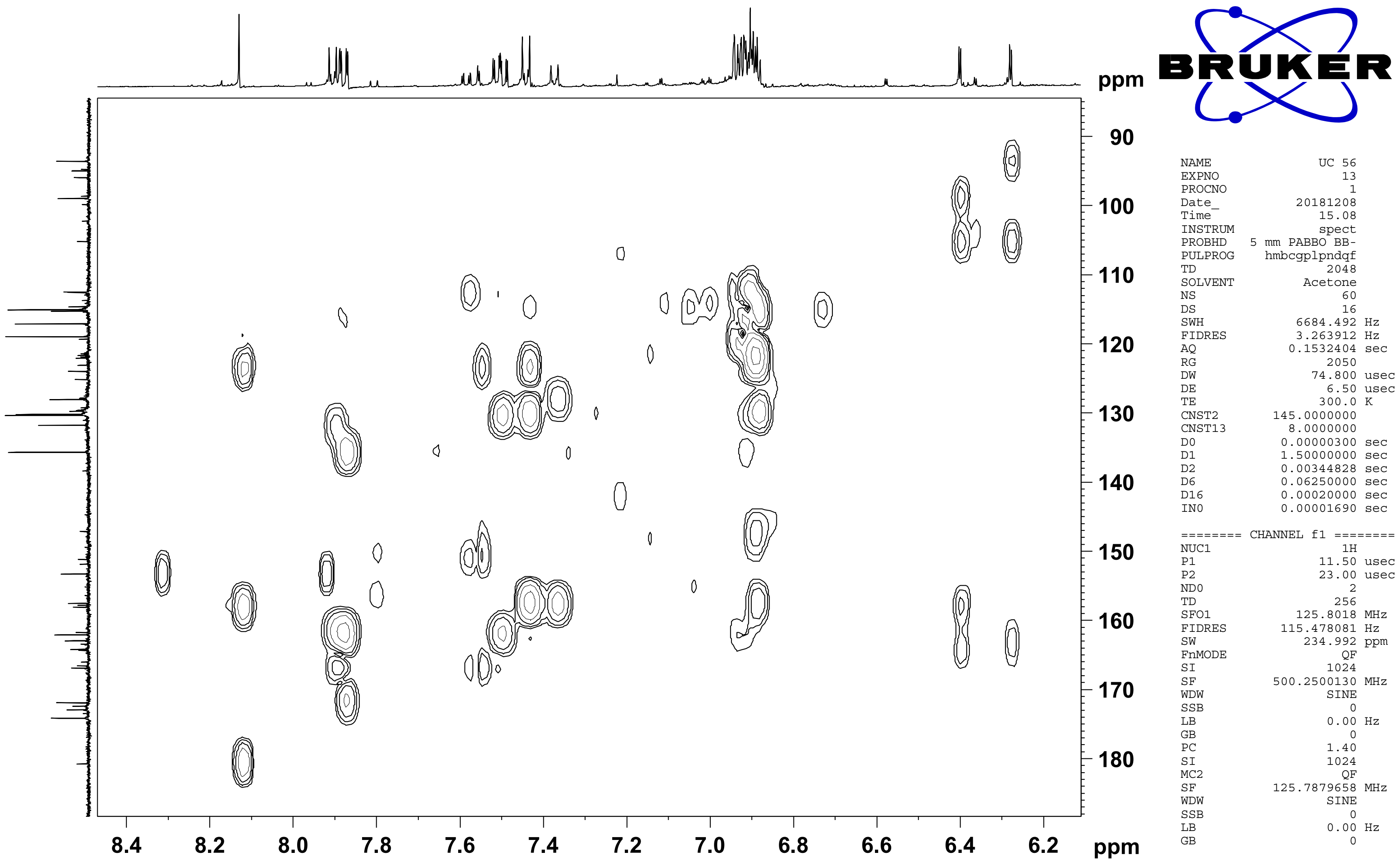

Fig. S3 Selected HMBC spectrum (acetone-d_6) of subfraction D-4.

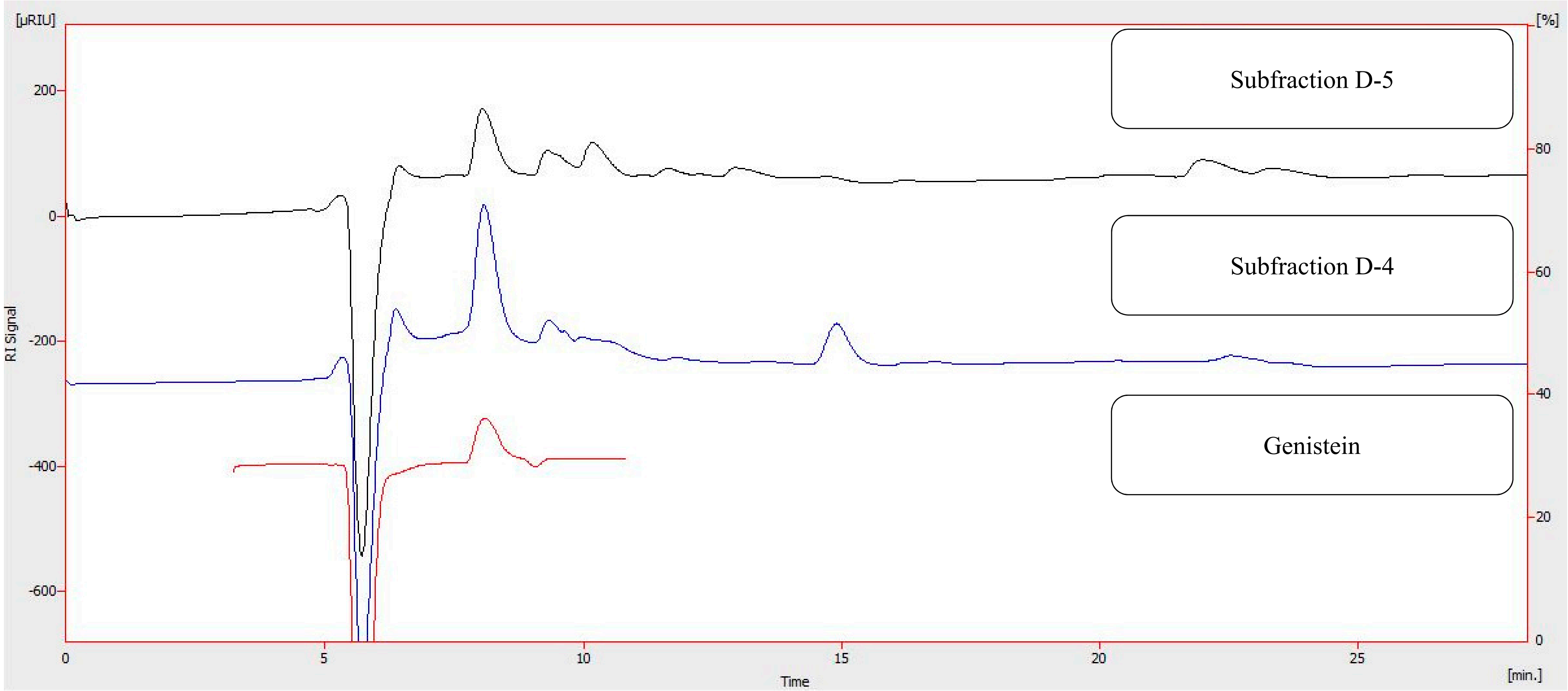

Fig. S4 Chromatogram of genistein-containing subfractions D-4 and D-5.

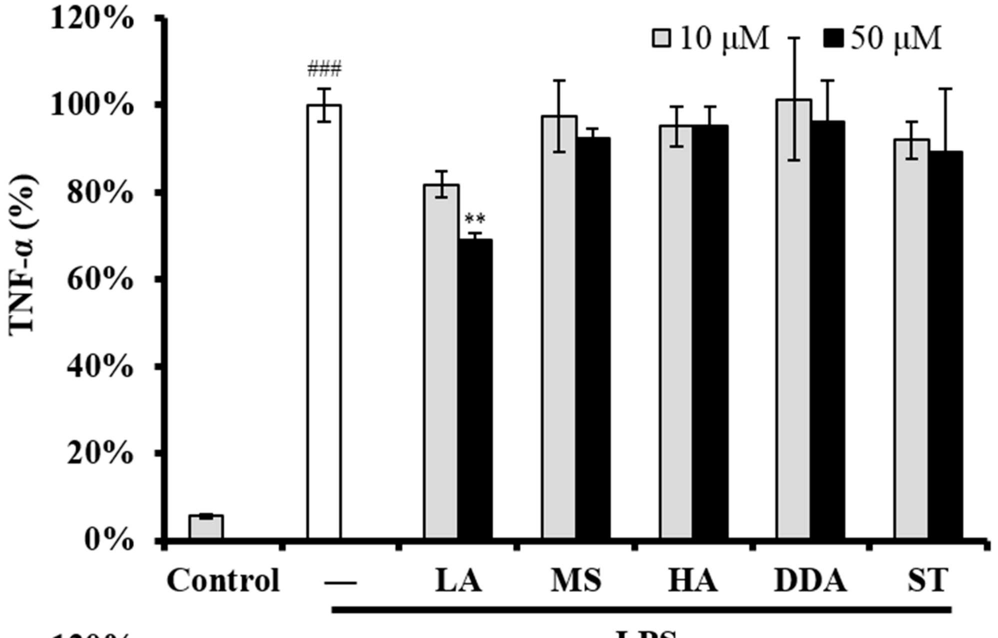

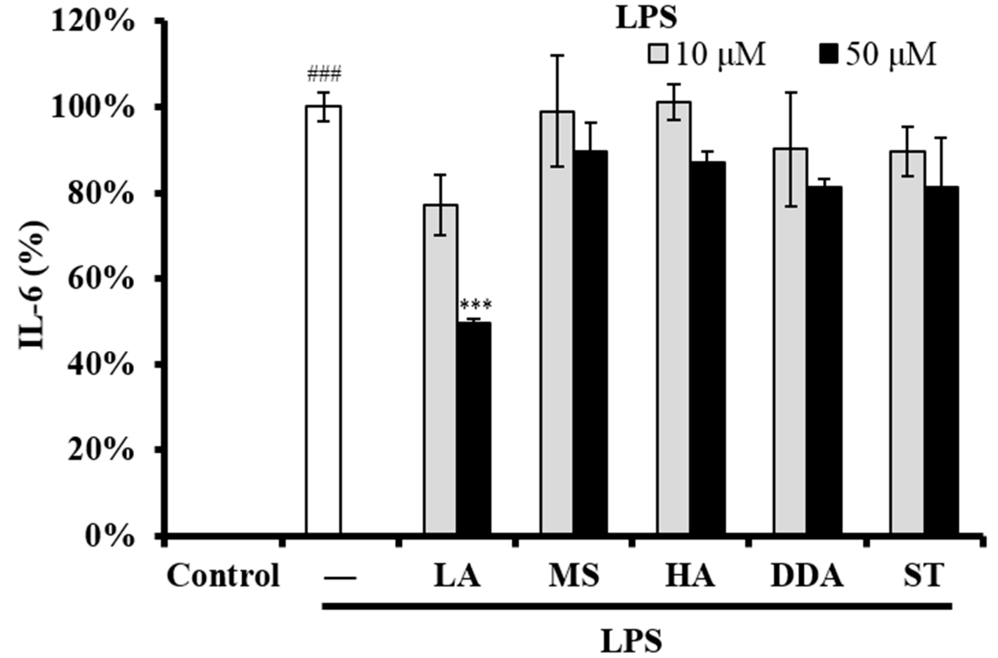

Fig. S5 The effects of the compounds LA (lupinalbin A), MS (*p*-hydroxybenzoic acid), HA (*p*-hydroxybenzoic acid), DDA (*p*-hydroxybenzoic acid), and ST (a mixture of *β*-sitosterol and stigmasterol) on the production of pro-inflammatory cytokines in LPS-stimulated DCs.

DCs were untreated or treated with LPS (100 ng/mL, white bar), LPS + LA, MS, HA, DDA, and ST (10 μM, gray bar or 50 μM, black bar) as indicated. Supernatants were collected at 6 h after the treatment. The production of cytokines (TNF-*α* and IL-6) were measured by ELISA and expressed as percentage inhibition. Data shown are the mean ± SD of three independent experiments. $^{###}p < 0.001$; $^{*}p < 0.05$; $^{**}p < 0.01$; $^{***}p < 0.001$ (Scheffe's test) are comparisons between sample-treated and non-treated LPS-stimulated DCs.

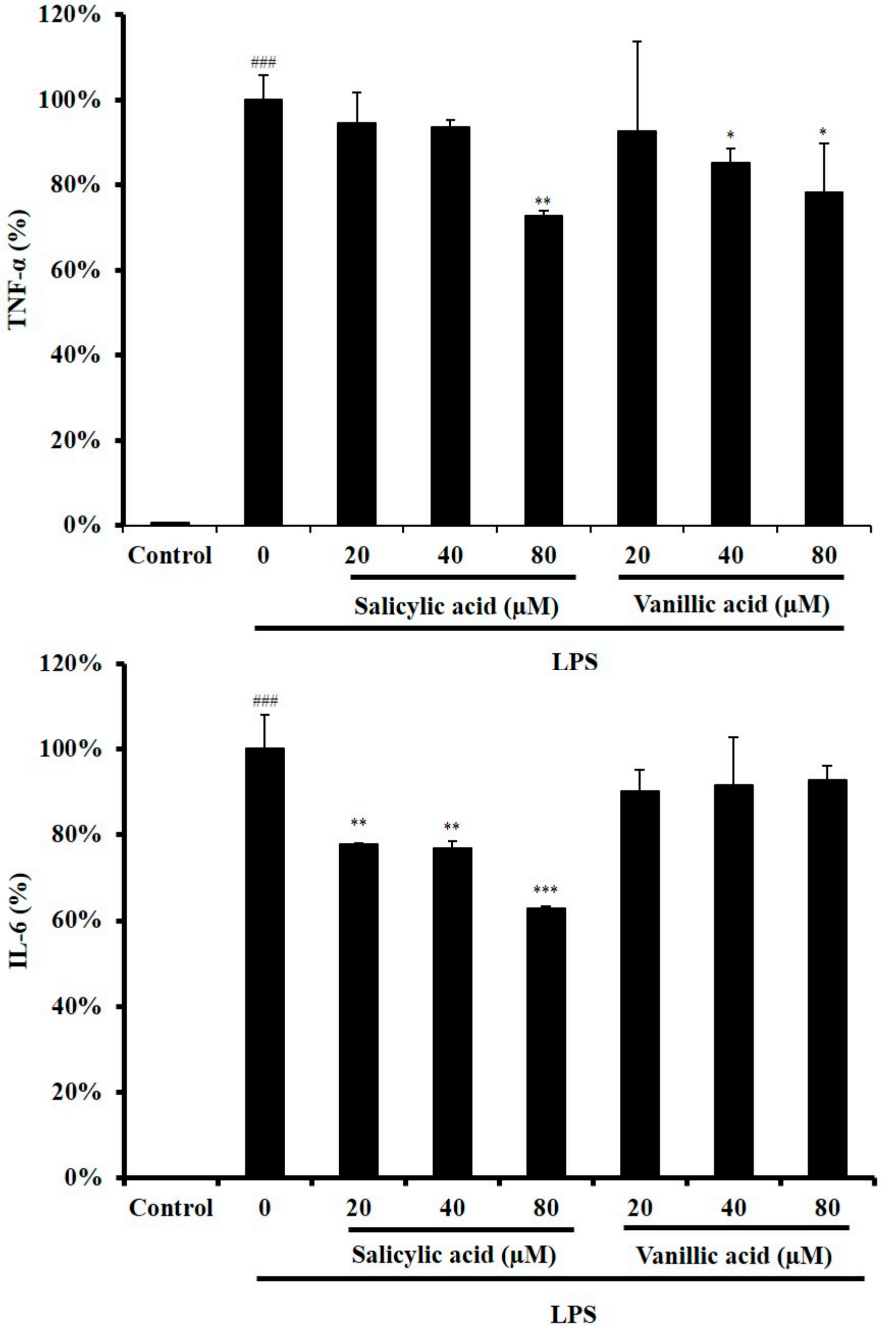

Fig. S6 The effects of the compounds SA (salicylic acid) and VA (vanillic acid) on the production of pro-inflammatory cytokines in LPS-stimulated DCs.

DCs were untreated or treated with LPS (100 ng/mL, white bar), LPS + SA and VA (20, 40, and 80 μM) as indicated. Supernatants were collected at 6 h after the treatment. The production of cytokines (TNF-*α* and IL-6) were measured by ELISA and expressed as percentage inhibition. Data shown are the mean ± SD of three independent experiments. $^{###}p < 0.001$; $^{*}p < 0.05$; $^{**}p < 0.01$; $^{***}p < 0.001$ (Scheffe's test) are comparisons between sample-treated and non-treated LPS-stimulated DCs.
